# Health Tract, No. 186

**Published:** 1864-02

**Authors:** 


### DERANGED.

INSANITY means literally without health as to the brain; its most common cause is the mind dwelling too much on one idea, or having a too great sameness of occupation, especially of an all-absorbing or unpleasurable character, as witness inventors, great geniuses, etc. The Superintendent of a State Lunatic Asylum states that the most furious maniac he had ever known was a woman who had raised a large family of children, each of whom was sent out to work as soon as able to do so, while she nursed the younger ones and did nearly all the work of the family herself; here was not only sameness of occupation, but an unpleasant sense of being driven all the time; anxiety, wearing care and solicitude pervading the whole of her existence. The insane are generally those who have had some great trouble; disappointed affection; loss of a dear relative or bosom friend; pecuniary reverses, or eating remorse. Had any one of these been called to encounter half a dozen troubles, each equal to the first, there would have been no derangement at all, because the nervous stream would have expended its force, or have been diverted to half a dozen different points instead of one, and thus would not have caused disorganization or an uncontrollable action as of the one over-stimulated portion. A man who thinks and talks incessantly of one thing, is soon set down by his neighbors as "crazy on that subject," although sensible enough on others. The fear of poverty has made many a rich man go mad. But the hardest worked slave is seldom deranged, because he has no abiding sorrow; no concern about to-morrow's bread; his labor is mechanical, and the moment it is over he dismisses all thought of toil, the mind runs home to his little hut, to his supper, and the other animal gratifications of his position, and his sleep is infinitely sweeter than his master's. In educated and elevated New-England there are nearly ten times as many crazy persons as among an equal number of field hands in the South. Taking planters and their slaves together, there are three times fewer insane than in as many New-Englanders. More crazy people come from the farm than from the city and the town, in spite of the coveted quiet of a farmer's life, its envied independence, and its wrongly estimated abundance of the good things of this life. There are not half as many deranged people in the Western States as in New-England, in proportion to the population. One general principle explains these varying conditions. New-England is thickly settled; its soil is sterile; its winters long and dreary, and the competition for bread is ceaseless and terrific; the mind frets at the long winter's inaction; it is like a caged lion; it beats unavailingly against its prison bars, and wastes itself in castle building and "vain thoughts." To be without money is to be without bread in New-England; in the sunny South and in the broad fields of the blooming West the people "take trust for pay," and can live for years on confidence and credit, and a fear for to-morrow's bread never enters the imagination. Ohio is a fertile State, but thickly settled; the two antagonize each other to some extent, so that the number of her lunatics is half-way between those of New-England and the West. Therefore, divert the mind in time of trouble; don't brood over misfortunes, nor indulge in melancholy meditations; gloat not over gold; never allow your reflections to become inseparable from any one subject. When you find that you "can't sleep" from the mind running on a particular subject, remember that you are rapidly preparing for the mad-house, and in proportion as any one idea absorbs the brain, in such proportion are you courting insanity. Cultivate a cheerful, an uncomplaining, a genial frame of mind. Look on the bright side of things; take hold of the smooth handle; and above all be moderately busy to the last day of life in something agreeable and useful to yourself and others.

